# Effect of Hot Isostatic Pressing on Porosity and Mechanical Properties of 316 L Stainless Steel Prepared by the Selective Laser Melting Method

**DOI:** 10.3390/ma13194377

**Published:** 2020-10-01

**Authors:** Tomas Cegan, Marek Pagac, Jan Jurica, Katerina Skotnicova, Jiri Hajnys, Lukas Horsak, Kamil Soucek, Pavel Krpec

**Affiliations:** 1 Faculty of Materials Science and Technology, VSB—Technical University of Ostrava, 70800 Ostrava, Czech Republic; jan.jurica@vsb.cz (J.J.); katerina.skotnicova@vsb.cz (K.S.); 2 Faculty of Mechanical Engineering, VSB—Technical University of Ostrava, 70800 Ostrava, Czech Republic; marek.pagac@vsb.cz (M.P.); jiri.hajnys@vsb.cz (J.H.); 3 Centre for Advanced Innovation Technologies, VSB—Technical University of Ostrava, 70800 Ostrava-Poruba, Czech Republic; lukas.horsak@vsb.cz; 4 Institute of Geonics of the Czech Academy of Sciences, Studentska 1768, 708 00 Ostrava-Poruba, Czech Republic; kamil.soucek@ugn.cas.cz; 5 V-NASS, a.s., Halasova 2938/1a, 703 00 Ostrava-Vítkovice, Czech Republic; pavel.krpec@v-nass.cz

**Keywords:** additive manufacturing, 316 L steel, selective laser melting, porosity, hot isostatic pressing, X-ray computed micro-tomography, tensile strength

## Abstract

The manufacturing route primarily determines the properties of materials prepared by additive manufacturing methods. In this work, the microstructural features and mechanical properties of 316 L stainless steel prepared by the selective laser method have been determined. Three types of samples, (i) selective laser melted (SLM), (ii) selective laser melted and hot isostatic pressed (HIP) and (iii) selective laser melted and heat treated (HT), were characterized. Microstructural analysis revealed that SLM samples were formed by melt pool boundaries with fine cellular–dendritic-type microstructure. This type of microstructure disappeared after HT or HIP and material were formed by larger grains and sharply defined grain boundaries. The SLM-prepared samples contained different levels of porosity depending on the preparation conditions. The open interconnected LOF (lack of fusion) pores were observed in the samples, which were prepared with using of scanning speed 1200 mm/s. The blowhole and keyhole type of porosity were observed in the samples prepared by lower scanning speeds. The HIP caused a significant decrease in internal closed porosity to 0.1%, and a higher pressure of 190 MPa was more effective than the usually used pressure of 140 MPa, but for samples with open porosity, HIP was not effective. The relatively high yield strength of 570 MPa, tensile strength of 650 MPa and low ductility of 30–34% were determined for SLM samples with the lower porosity content than 1.3%. The samples after HIP showed lower yield strengths than after SLM (from 290 to 325 MPa) and relatively high ductility of 47.8–48.5%, regardless of the used SLM conditions.

## 1. Introduction

Additive manufacturing (AM) methods, which work on the principle of layer by layer, represent powerful freeform fabrication techniques which can fabricate direct deployable components without the necessity of special machining, and are highly efficient when only small quantities are required [1,2,3,4,5]. Selective laser melting (SLM) is the most commonly used AM method for forming metal parts, where the starting material is in pre-alloyed powder form [2]. However, the mechanical properties of the materials prepared in this way are significantly affected by the preparation conditions (energy density) and also show a significant anisotropy with respect to the load axis during mechanical testing and the direction of preparation [1,2,3,4]. Hitzler [1], for example, states that SLM stainless steel possesses its maximum strength at a 45° layer versus loading offset. Although SLM is able to manufacture almost fully dense parts (98–99%), the presence, as with all powder-based processes (sintering, hot isostatic pressing) and as with other net-shape manufacturing methods (casting), is an inherent porosity associated with the process. Similar to conventionally manufactured parts, the residual porosity hinders high-strength and fatigue resistance applications [3]. Three types of the porosity have been defined to date. The first type of porosity is caused by insufficient or imperfect melting of particles (lack of fusion (LOF) porosity), exhibits angular and random morphologies with a large size of up to 500 µm and usually contains unmelted powder [6]. LOF porosity arises due to the small variance in the energy density of the laser across the surface of the layer and can be partially corrected by changing the process parameters. The other two types of porosity arise due to gas entrapment by surface turbulence. Blowhole porosity is characteristic by an oval shape and small size of up to ten micrometers, and the third type is keyhole porosity, which arise from the collapse of keyhole walls usually at higher laser intensity, and it is characteristic by larger dimensions than blowhole porosity [6,7,8]. In the case of turbulent capturing the gas, where evaporation of the material or gas of the protective atmosphere leads to porosity, it can be very difficult to correct [9]. The present article is aware of this fact, and one of the main objectives of this article is the reduction or removal of the internal porosity of the steel printed parts using hot isostatic pressing (HIP) as post-process technology. This technology, due to high pressure, temperature and sufficient time, allows the material to flow together in a solid state to form bonds at the atomic level, thus changing the microstructure and reducing porosity [2,10]. The investigated material was 316 L austenitic steel, which is the most widely used material in the medical implant sector and is commonly used in the chemical and petrochemical industry, food processing and others [11]. Although the influence of HIP on the porosity and mechanical properties of 316 L steel after SLM preparation has already been studied in some detail in several publications, the results are not entirely consistent. Lavery [2], for example, states that after SLM of 316 L steel, irregular and highly directional porosity was observed when using a lower energy density, and a smaller, more rounded and randomly distributed porosity was observed when using a higher laser density. Additionally, in both cases, the porosity was reduced by hot isostatic pressing. On the other hand, Rottger [4] states that no significant reduction of porosity could be achieved during HIP post treatment of 316 L SLM-prepared parts, whereas the samples before HIP showed similar contents of porosity of 0.3–4% and 2%, respectively, as well as similar HIP conditions of 1125 °C/137 MPa/4 h and 1150° C/150 MPa/3 h. However, both authors used different laser powers of 180 and 100 W and different exposure times for SLM and HIP samples of 70–150 and 400 µs, respectively. Thus, these publications suggest that the effect on the elimination of porosity during HIP post processing is affected by the conditions used during SLM, and in the case of using a laser with lower powers and short exposure times, HIP may not be effective to eliminate residual porosity. Montero-Sistiaga [5] states that when using a laser with a power of 400 and 1000 W for preparation of samples from 316 L steel, HIP was effective to eliminate porosity and cracks, while the conditions of HIP were similar to those of Rottger, namely 1155 °C/100 MPa/3 h. Additively manufactured 316 L stainless steel usually requires post-processing heat treatment (recovery, homogenization, annealing) to maximize strength and ductility. The application of heat treatments removes the melt pool boundaries and microsegregation in the as-printed material [12,13,14]. Those microstructural differences influence mechanical properties such as resilience behavior [15,16]. However, despite the improvement of the mechanical properties, the heat treatment process is not presently designed to remove any porosity inherent in the SLM process.

The aim of this article is to primarily test the different laser power, exposure times and scanning strategy (meander, chessboard) during the preparation on the microstructure and distribution of porosity in the samples, and to describe the effect of HIP on the elimination of porosity, microstructural changes and mechanical properties of 316 L steel. In addition, another aim of the article is to determine the effect of the higher applied pressure (190 MPa) during HIP, because for almost all available publications concerning HIP of SLM parts of 316 L steels, a maximum pressure of 150 MPa was used [2,4,5,12]. A prediction is that higher pressure can be significantly more effective for elimination of porosity during HIP. A positive effect of higher pressure is also indicated by the results published by Puichaud [17], who reports the elimination of nanoscale porosity during HIP at 180 MPa in 316 L steel samples after SLM.

## 2. Materials and Methods

### 2.1. Starting Powder and Powder Analysis

SS 316 L-0407 stainless steel atomized powder provided by Renishaw was used for this study. Besides the SEM analysis of the particle shape and microstructure and EDS analysis of chemical composition, the powder size and size distribution were evaluated by means of a MasterSizer 3000 laser diffraction particle size analyzer (Malvern Panalytical Ltd., Malvern, UK). The amount of oxygen, nitrogen and carbon, sulfur were measured by the thermo-evolution method using the ELTRA ONH-2000 and ELTRA CS-2000 (Eltra GmbH, Haan, Germany) analyzer devices, respectively. Samples for thermo-evolution analysis with dimensions of 5 mm × 5 mm × 5 mm were prepared by cold isostatic pressing using the Engineered Pressure Systems International nv (EPSI, Temse, Belgium) cold isostatic presser and pressure of 350 MPa for 30 s.

### 2.2. Selective Laser Melting (SLM)

**Figure 1:**
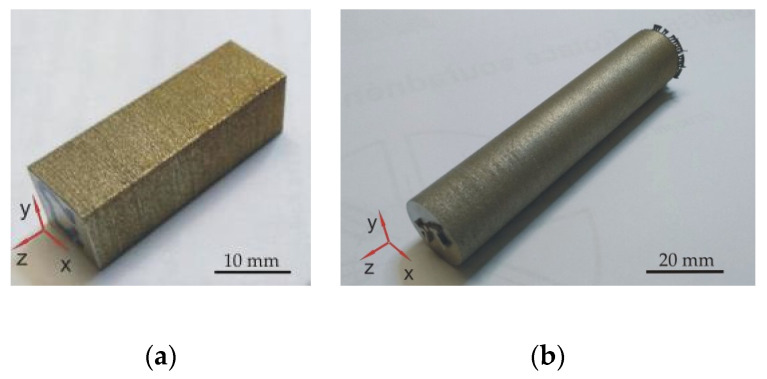
(**a**) selective laser melting (SLM)-prepared rectangular stainless steel sample 5 and (**b**) SLM-prepared cylindrical stainless steel sample 2.

**Figure 2:**
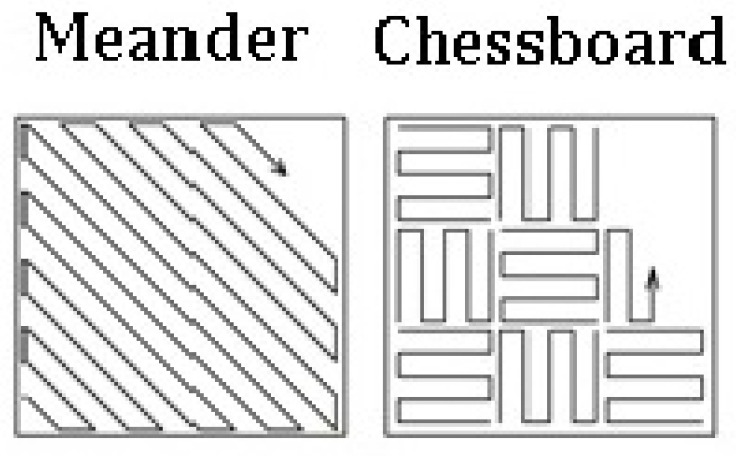
Used scanning strategies [21].

SLM densification was performed using the Renishaw AM400 device (Wotton-under-Edge, Great Britain). Sixteen different combinations were selected for preparation of 10 mm × 10 mm × 38 mm rectangular stainless steel samples designed for microstructural analysis and measurement of density and porosity (48 pieces total, 3 for each batch). The experiment was guided by the Taguchi Orthogonal Array Design L16 (4**2 2**1) [18], as well as, for example, Yang [19] for the study of alloy Ti-6Al-4V prepared by SLM. An example of the prepared sample is shown in Figure 1a. The samples were prepared layer by layer in the vertical direction (z axis). Different laser outputs, scanning speeds and layer formation strategies were included (see Table 1). The other process parameters, namely, hatch distance (0.11 mm), spot diameter (70 µm), and layer thickness (50 µm), were constant. Substrate preheat was not applied. The Renishaw AM400 device uses a pulsed laser, so it was necessary to use an exposure time calculation to determine the effective scan speed. For determination of the effective scanning speed ν (mm/s) Equation (1) was used [20]:

υ = 60/(ET + 12) × 10^3^ (1)

where ET is exposure time (µs). The chosen strategies, namely, meander and chessboard (see Figure 2), have different uses and benefits. Meander, the most common strategy, works on the principle of scanning and gradual alternation of layers with mutual orientation and on rotating the new layer over the previous layer by an angle of 67°. As a result of this, it reaches the same direction again after the 180th layer. This method reduces the occurrence of porosity and is recommended for components with smaller XY cross sections. It is fast and efficient. The disadvantage is that this strategy involves inconsistent heat distribution in each layer. The chessboard strategy works on the principle of dividing individual fields (like a chessboard) into a size of ~5 mm^2^, which are rotated on each other by 90°. To avoid porosity, field offset is used in this strategy to overlap the fields. The advantage of this strategy is that due to the division into individual fields, the heat is not very high at one point, but the strategy is significantly slower than the others [20].

Other sets of 54 cylindrical tensile test samples with use of 6 different laser outputs, scanning speeds and layer formation strategies (1, 2, 5, 6, 9, 13 from Table 1) were printed in parallel using the same conditions as during the printing of rectangular samples. The cylindrical samples diameter was 20 mm, its length was 100 mm, and the shape and size of the sample were designed for easy preparation of specimens for mechanical tests (see Figure 1b). The samples were prepared in the vertical direction as well as the rectangular direction.

### 2.3. Hot Isostatic Pressing (HIP)

**Figure 3:**
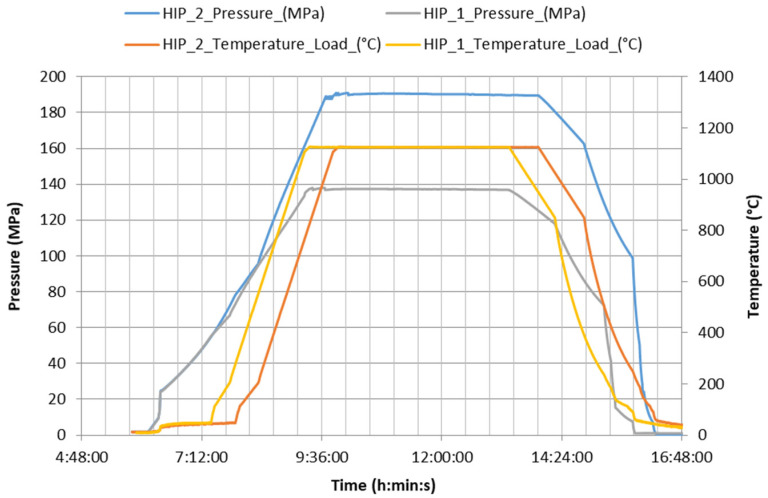
Temperature and pressure during hot isostatic pressing (HIP) processes.

For the investigation of the effect of HIP on the porosity elimination and on the mechanical properties, 16 rectangular specimens prepared by 16 different conditions (samples 1–16, see Table 1) were HIPed at 1125 °C/4 h/137 MPa (HIP_1 conditions in text) at EPSI (Temse, Belgium) hot isostatic presser. After dwell time at the pressure and temperature controlled cooling 5 °C/min was used to 850 °C and furnace cooling at lower temperatures. Another 6 rectangular specimens (samples 1, 2, 5, 6, 9, 13 from Table 1) and 18 cylindrical test samples prepared by 6 different conditions of SLM (samples 1, 2, 5, 6, 9, 13 from Table 1, 3 for each batch) were HIPed at higher pressure 190 MPa (HIP_2 conditions in text) with using of the same temperature, dwell time and cooling conditions. The temperature and pressure records for both processes are shown in Figure 3. Argon with purity 99.96% was used during HIP. No capsule or sealed envelope was used during the HIP.

### 2.4. Heat Treatment

Another 18 cylindrical samples after SLM (samples 1, 2, 5, 6, 9, 13 from Table 1 and Table 2 for each batch) were heat treated at the same temperature, dwell time and cooling rates as HIP samples to compare the effect of pressure during HIP on mechanical properties of SLM samples. Heat treatment was performed in a tube furnace in a dynamic atmosphere of Ar with a purity of 99,995%.

### 2.5. Metallography, Phase Identification and Microscopy

Samples for metallographic investigation were cut from the central part of SLM samples by electro-erosion cutting (cutting in running water at a voltage of 1.8 kV and a current of 150 mA). The cut was made parallel to the xy direction. Standard metallographic techniques, including grinding on SiC papers with grain sizes ranging from 60 to 2000 (grains/cm^2^) and polishing with Al_2_O_3_ suspension with particle size changing from 1 to 0.3 µm, were applied. The samples were studied by optical microscopy (OM) on an Olympus GX 51 (Olympus Corporation, Shinjuku, Japan) microscope and scanning electron microscopy (SEM) in back-scattered electron (BSE) mode using a Quanta 450 FEG microscope (FEI Company, Fremont, CA, USA) equipped with an energy-dispersive X-ray spectrometer (EDS). XRD analysis was carried out using a Bruker D8 DISCOVER diffractometer (Bruker, Billerica, MA, USA) equipped with an X-ray tube with a rotating Cu anode operating at 12 kW. All measurements were performed in parallel beam geometry with a parabolic Goebel mirror in the primary beam. Diffraction patterns were measured within an angular range of 20–70° of 2 Ɵ with an exposition time of 5 s and step size of 0.05°. The Rietveld method was used to estimate the amount of phases.

### 2.6. Density and Porosity Measurement

The helium pycnometry method was used for determination of the density (ρ_pycno_) of SLM and SLM/HIP samples using a gas pycnometer AccuPyc II 1340 (Micrometrics Ltd., Doddington, UK) with an integrated analysis module. Based on measurements on a gas pycnometer, the closed porosity P_cl_ was determined according to the relationship:

P_cl_ = (1 − ρ_pyc_/ρ_teo_) × 100 (2)

where ρ_teo_ represent theoretical density of 316 L steel. Published values of the theoretical density of 316 L steel in the range of 7.95–8.00 g/cm^3^ [2] were used. The resulting density was determined based on 10 cycles for each measured sample.

### 2.7. Optical Image Analysis to Determine Porosity Levels

Optical microscopy was used to observe the porosity of selected polished samples after SLM and SLM + hot isostatically pressed samples. For each sample, the central parts of the sample cross-sections were imaged at 100× magnification and sufficient pixel resolution to establish porosity above 2 µm. Image analysis was undertaken using the ImageJ software (Madison, WI, USA). Porosity levels were determined by adjusting the brightness threshold, and porosity fractions were calculated automatically on the basis of the contrast between dark porosity and bright solid material. The five different areas were analyzed with the distribution of individual analyzed areas from edge to edge of the samples in the x direction with a distance 2 mm.

### 2.8. X-ray Computed Microtomography

A non-destructive method of industrial X-ray-computed microtomography was used to study the comparability of two rectangular samples (5–SLM, 5–SLM + HIP_2). An NV XT H 225 ST (Nikon Metrology, Brighton, MI, USA) tomograph was used for scanning of tomographic volumes, and 3D-CT Pro (Nikon Metrology, Brighton, MI, USA) software was used for the reconstruction of the tomographic data. VGSTUDIO MAX 3.3.2 software (Volume Graphics GmbH, Heidelberg, Germany) was applied for analysis of the porosity and internal construction of scanned samples. The test specimens were scanned at an accelerating voltage of 220 kV and at a power of 99 W with the size of the focal spot at about 80 µm. For the CT reconstruction, radiographic projections of 3141 volume scanned during rotation of samples by 360° were used. The time required to capture one radiographic projection took about 22 s. The CT scanning and reconstruction process of one sample takes approximately 22 h, and the resulting size of the individual cubic voxels in CT volume is represented by a value of cc. 8 µm (the voxel resolution is directly proportional to the geometrical magnification of the sample on the flat panel X-ray detector). The analyzed height of both tested specimens was 11 mm.

### 2.9. Tensile Testing

**Figure 4:**
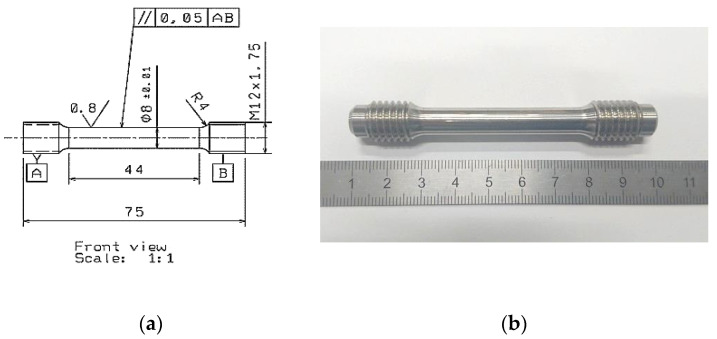
(**a**) Geometry of used tensile test samples and (**b**) SLM sample prepared for tensile testing.

The mechanical behavior of the cylindrical samples (SLM, SLM + HIP_2, SLM + heat treated (HT)) was evaluated by tensile testing. Cylindrical tensile specimens were built layer by layer in vertical z direction and lathe machined to the final shape with using of ST-10 device (HAAS, Oxnard, CA, USA) (see Figure 4). The specimens were tested in an Instrom Z 150 (Instrom, Ulm, Germany) device with a cross-head velocity 0.5 min^−1^. Three tensile tests were performed for each batch of samples.

## 3. Results and Discussion

### 3.1. Powder Particle Analysis

**Figure 5:**
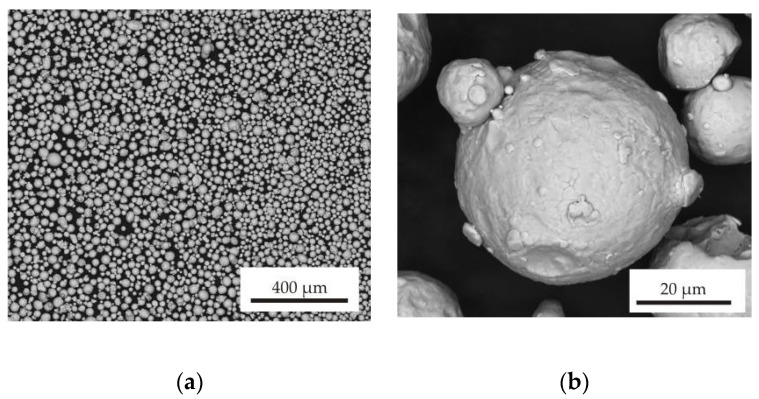
Scanning electron microscopy (SEM) in back-scattered electron (BSE) mode micrographs of atomized powder: (**a**) 200× magnification (**b**) 5000× magnification.

**Figure 6:**
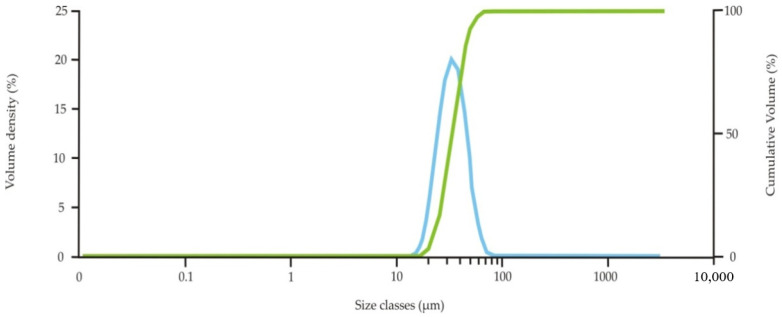
Particle size distribution diagram of the used 316 L powder.

**Figure 7:**
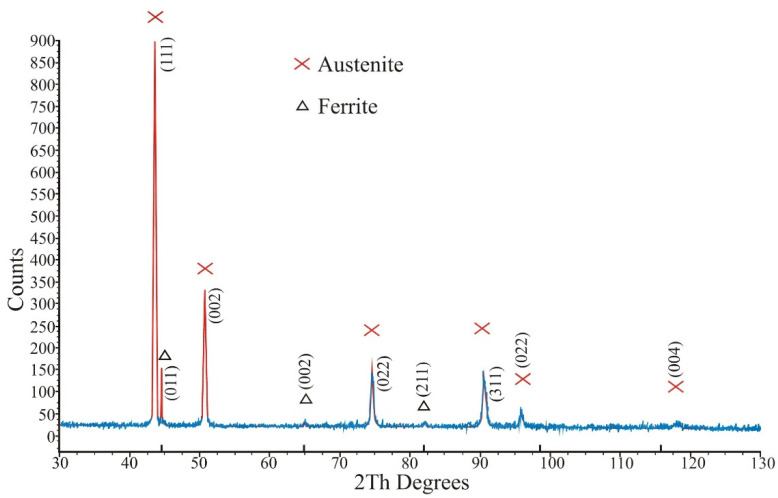
X-ray diffraction patterns of the used powder.

SEM BSE micrographs of the used 316 L powder particles are shown in Figure 5. The particles possessed a spherical shape, which is typical for powders prepared by gas atomization. Particle size distribution is shown in Figure 6. The mean particle diameter was 33.7 µm, and the span corresponded to a value of 26 µm. Several satellites (smaller particles of up to 10 µm adhered to larger particle surfaces) were observed mainly on larger powder particles (see Figure 5b). The presence of satellites on powder surfaces reduces flowability and affects powder packing [22]. Table 3 shows a comparison of the chemical composition of the powder declared by the manufacturer and the determined composition by the EDS method. The determined chemical composition is therefore in accordance with the nominal composition as well as the contents of interstitial elements whose contents were lower than those reported by Renishaw, and its content were about 450, 250, 200 and 40 wt. ppm for O, N, C and S respectively. Rietveld analysis of the XRD diffraction pattern revealed that the powder contained 96 vol.% of austenite and 4% of ferrite. The XRD diffraction diagram of powder is shown in the Figure 7.

### 3.2. Microstructure of SLM Samples

**Figure 8:**
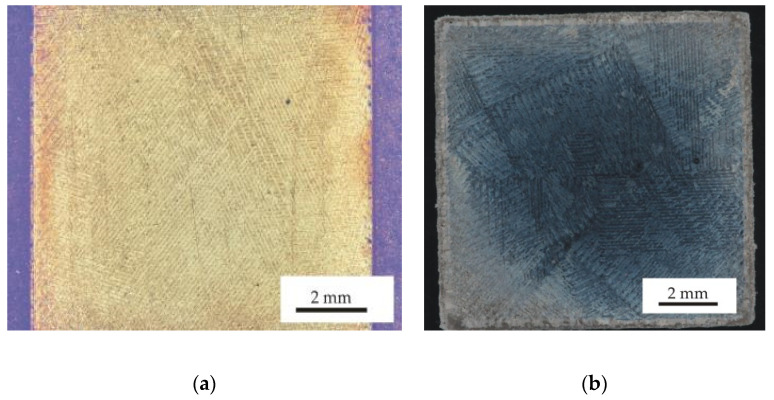
(**a**) Optical microscopy OM image of sample 2 after SLM and (**b**) OM image of sample 9 after SLM, etched with Carpenter reagent.

**Figure 9:**
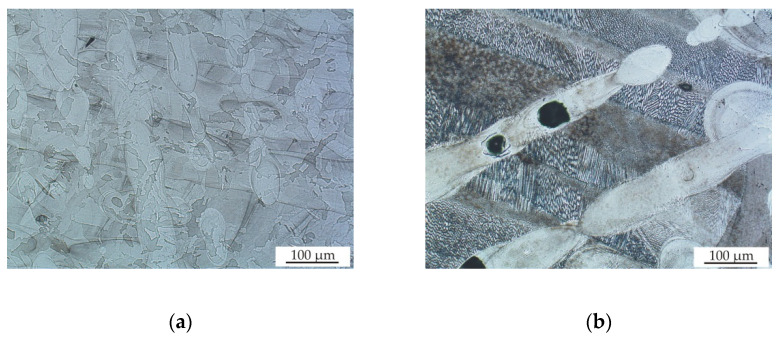
(**a**) OM image of sample 2 after SLM and (**b**) OM image of sample 13 after SLM, etched with Carpenter reagent.

**Figure 10:**
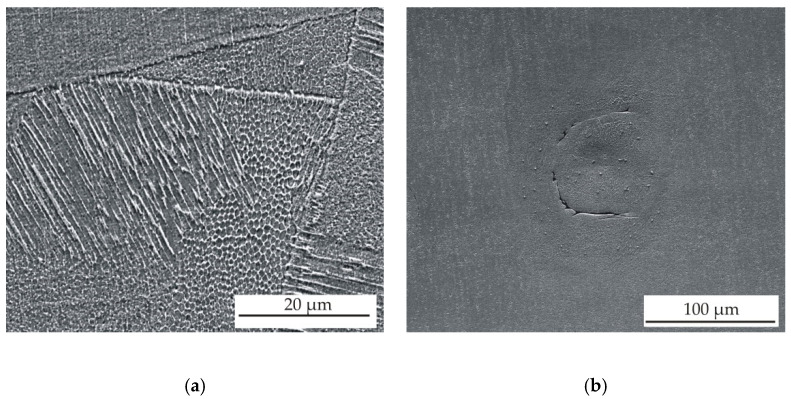
(**a**) SEM image of sample 5 after SLM and (**b**) SEM image of defect in sample 6, etched with Carpenter reagent.

**Figure 11:**
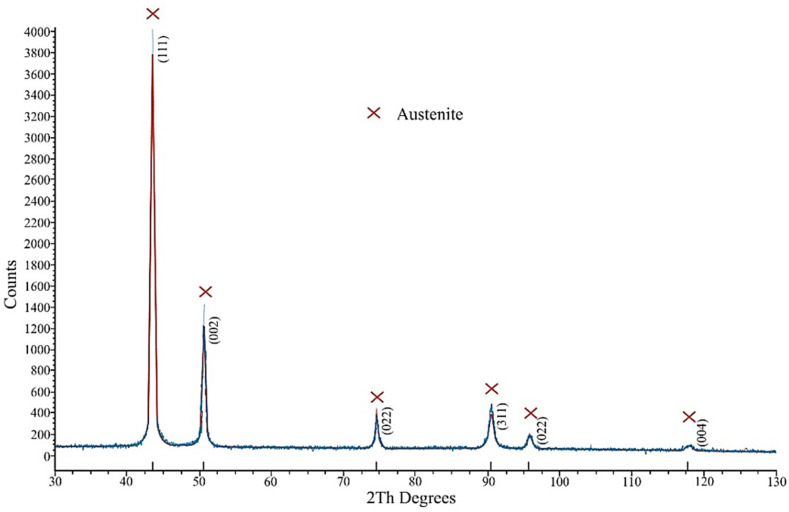
X-ray diffraction patterns of sample 6 after SLM.

Figure 8 shows the cross-sectional views on the x–y scan direction plane for the samples 2 and 9, which were prepared by meander and chessboard strategies, respectively. Typical scan tracks with an angle of 67° between layers (see Figure 8a) and a distribution of sample area into individual fields with different scan directions (see Figure 8b) can be observed, which is typical for the meander and chessboard scan strategies, respectively. These melted scan tracks are representative of the solidified melt pool for each layer on the powder bed. Figure 9a,b show the microstructure of the prepared samples at higher magnifications. The typical over-lapping geometries can be clearly observed, which demonstrate successful fusion of powder particles and bonding within each layer, similar to the works [3,23,24]. A fine cellular–dendritic microstructure could be observed for all SLM-prepared samples (see Figure 10a), which is typical for steels prepared by SLM due to the short laser and material interaction and rapid solidification rates in the locally melted areas [3]. However, a significant proportion of porosity and defects (see Figure 10b) was found in the microstructure during the OM and SEM observations. Rietveld method analysis of XRD diffraction patterns revealed that samples after SLM contain only austenite, and the ferrite content is below the detection limit, at about one-tenth of a percent (see Figure 11).

**Figure 12:**
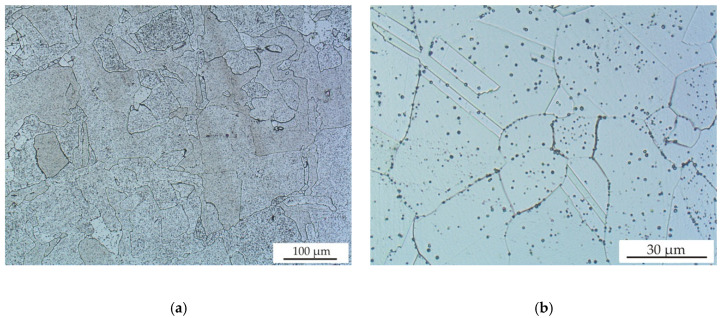
(**a**) OM image of sample 13 after HIP_1 treatment and (**b**) OM image of sample 2 after HIP_1 treatment, etched with Carpenter reagent.

Figure 12 shows the microstructure of samples after HIP. A more homogenous microstructure with larger grains and sharply defined grain boundaries can be clearly observed. These microstructures are consistent with the lower cooling rates during HIP. Microstructural observations at higher magnifications also revealed fine Si-rich oxides (small amount of Mn and Cr were also detected by EDS) mainly on grain boundaries and also inside the grains. The same fine oxides were also observed by Lou [25] in 316 L steel samples after SLM and HIP. Samples after SLM and HT displayed the same microstructure as after SLM and HIP, because the same temperature, dwell time and cooling conditions were used. Both samples after HIP and after HT contained only austenite and a minimal proportion of ferrite (below the detection limit of XRD diffraction) as well as after SLM. No significant differences in microstructure were observed for samples after HIP_1 and HIP_2 treatments.

### 3.3. Porosity

**Figure 13:**
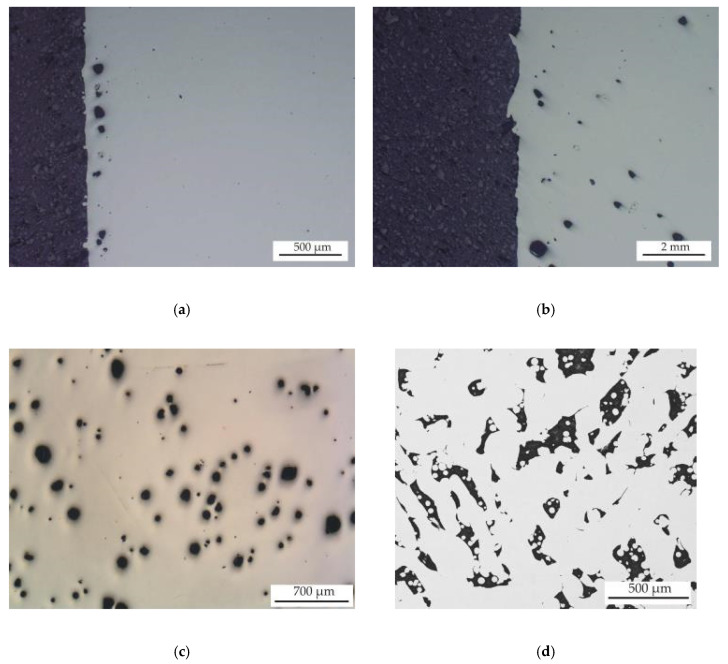
(**a**) OM image of sample 2 (scanning speed: 650 mm/s); (**b**) OM image of sample 5 (scanning speed: 400 mm/s); (**c**) OM image of sample 13 (scanning speed: 400 mm/s); (**d**) BSEM image of sample 4 (scanning speed: 1200 mm/s). All samples are after SLM and in a polished state.

As previously mentioned, in the case of 316 L steel after SLM, there is always a certain proportion of porosity, and the content of which significantly depends on the used conditions [2,5,6,9,12]. Figure 13 shows the different levels of porosity in the structure of the samples prepared under different SLM conditions. As can be seen, using a scanning speeds lower than 1200 mm/s caused randomly distributed rounded porosity, which was detected mainly at the edges of the samples (see Figure 13 a–c). Observed rounded porosity included both larger pores if up to 600 µm (keyhole-type porosity) and smaller pores with dimensions of up to 10 µm (blowhole-type porosity). The use of higher scan speeds of about 1200 mm/s in turn caused irregular LOF porosity, which was observed in the whole volume of the samples, in full accordance with Lavery assertion [2]. Table 2 includes the detected density values and the porosity values that were calculated on the basis of the results of the density measurements by the pycnometric method and results of porosity obtained by image analysis (ρ_image_). As can be seen in this table, individual methods of detecting porosity show quite different results. For example, the range of porosity determined by the helium pycnometric method was from 0.22% for sample 2 to 2.04% for sample 13, while the values of porosity determined by image analysis reached 0.13% for sample 2 and 18.7% for sample 4. These differences between the individual methods are caused by different contents of closed and open porosity in the samples. Porosity detected by image analysis includes both closed and open porosity, whereas pycnometric porosity includes only closed porosity, into which no gas enters. Only samples 4, 8, 12 and 16, which were prepared with a scan speed of 1200 mm/s, showed a high difference in porosity results determined by image analysis and the pycnometric method. This means that only the LOF pores in the samples prepared at a high scan speed of 1200 mm/s were open interconnected pores and in almost the entire volume, as shown in Figure 13d. These conclusions also indicate the determined lower volumes for these samples detected by pycnometric measurement, despite the same sample dimensions used during SLM. The highest porosity (P_image_) and the lowest pycnometric volume (V_pyc_) were observed in sample 4, which was prepared using a laser power of 200 W and a scanning speed of 1200 mm/s. With increasing laser power, the porosity for samples prepared with 1200 mm/s reached lower values of up to 5% of porosity in sample 16, which was prepared with laser power of 350 W. Samples 2, 6, 10 and 14 contained the lowest porosity levels of from 0.2 to 0.35% (detected by both used methods), which means that the most suitable used scanning speed was 650 mm/s. Samples prepared at a speed of 800 mm/s using a laser power of at least 250 W also showed low porosity (from 0.3 to 0.4%). It can also be stated that with increasing laser power, the porosity reached similar values for different speeds. The influence of the scanning strategy on the amount of porosity towards the scanning speed was minimal. It is necessary to note that density and porosity values in the Table 2 are the average of three results obtained by measuring the rectangular samples and from 9 results (12 total) of cylindrical samples for batch 1, 2, 5, 6, 9 and 13. For the remaining batches and for volumes, the values are the average of the measurements for three rectangular samples.

### 3.4. Effect of HIP on Porosity

**Figure 14:**
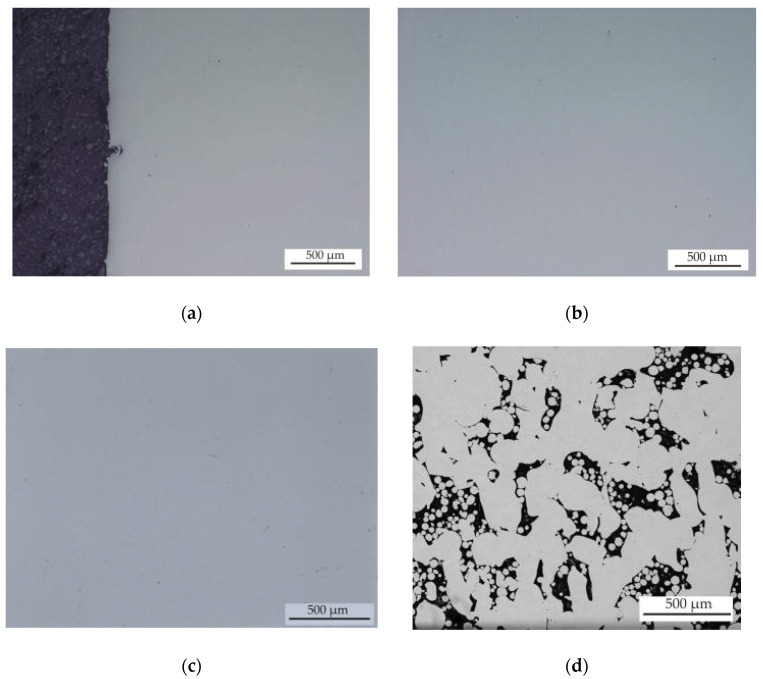
(**a**) OM image of sample 2; (**b**) OM image of sample 5; (**c**) OM image of sample 13; (**d**) BSEM image of sample 4. All samples are after the HIP_1 process and are in a polished state.

Figure 14 shows OM and BSEM images of samples after HIP at 1125 °C/4 h/137 MPa. A comparison of closed porosity values established by pycnometer and porosity values determined by image analysis in samples before and after HIP is present in Table 4. The table and Figure 14 clearly shows that HIP has a significant impact on the eliminating of internal closed porosity of SLM samples, but only a small effect on open porosity. For all samples, the volume fraction of closed porosity decreased to values less than 0.3%. The exceptions are samples 4, 8, 12 and 16, which contained the highest proportion of open irregular LOF porosity, which was created due to a high scan speed during SLM. During the HIP process, Ar enters into open pores; thus, these pores cannot be removed by plastic deformation during the HIP process. The samples with the majority of closed porosity containing blowhole- and keyhole-type porosity allowed a significant decrease in porosity after HIP. However, it should also be noted that in all samples, the elimination of internal closed porosity was not completely confirmed, and pycnometric measurements revealed a closed porosity, usually from 0.1 to 0.3 vol.% in samples after HIP at 1125 °C/4 h/137 MPa. For this reason, the effect of higher pressure during HIP was tested on selected samples.

### 3.5. Effect of Pressure on Porosity

**Figure 15:**
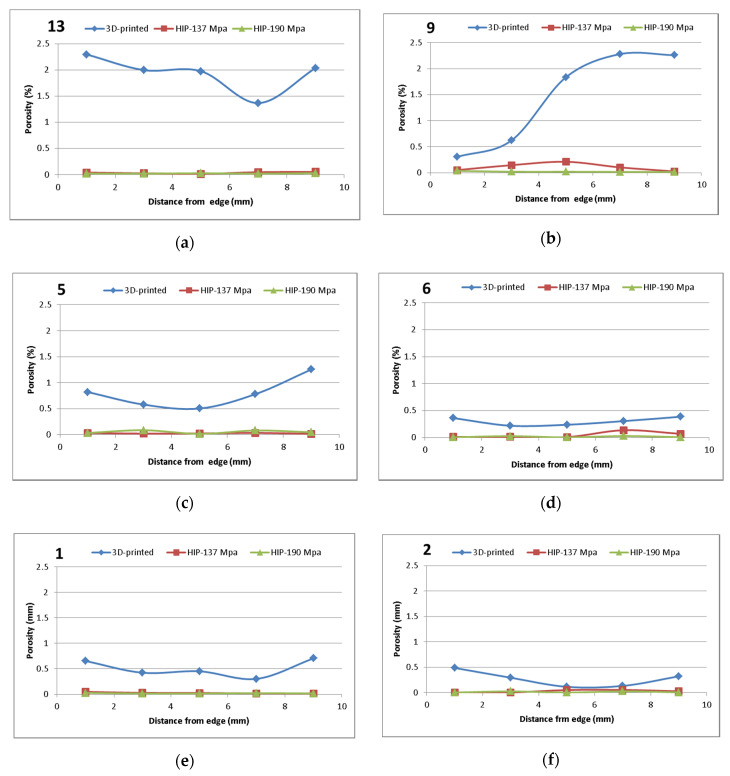
Porosity levels detected in samples after SLM, SLM + HIP_1 and SLM + HIP_2 by image analysis: (**a**) sample 13, (**b**) sample 9, (**c**) sample 5, (**d**) sample 6, (**e**) sample 1, and (**f**) sample 2.

The pycnometric density and closed porosity detected in the samples 1, 2, 5, 6, 9, and 13 after SLM, SLM + HIP at 1125 °C/4 h/137 MPa and SLM and HIP at 1125 °C/4 h/190 MPa are summarized in Table 5. All samples after HIP_2 process showed an internal closed porosity content of up to 0.11% and a pycnometric density of 7.94–7.95 g/cm^3^. For all samples, it can be stated that HIP had a significant effect on the reduction of closed porosity and on densification, and the use of higher pressure increased the efficiency of porosity elimination. SLM and SLM + HIP samples were also subjected to optical image analysis to assess the effect of HIP on porosity by this method. The porosity was determined after cutting the middle part of the samples and metallographic preparation. The results are shown in Figure 15. Optical image analysis found slightly different values of porosity to density measurements. However, this is due to the fact that only the middle part of the samples in one section was analyzed, while pycnometric measurements covered the entire volume. However, image analysis also confirmed a significant decrease in closed porosity in all samples after HIP and a higher efficiency of higher pressure during the hot isostatic pressing. Image analysis also suggests a higher proportion of porosity at the edges of the samples after SLM.

### 3.6. X-ray-Computed Microtomography

**Figure 16:**
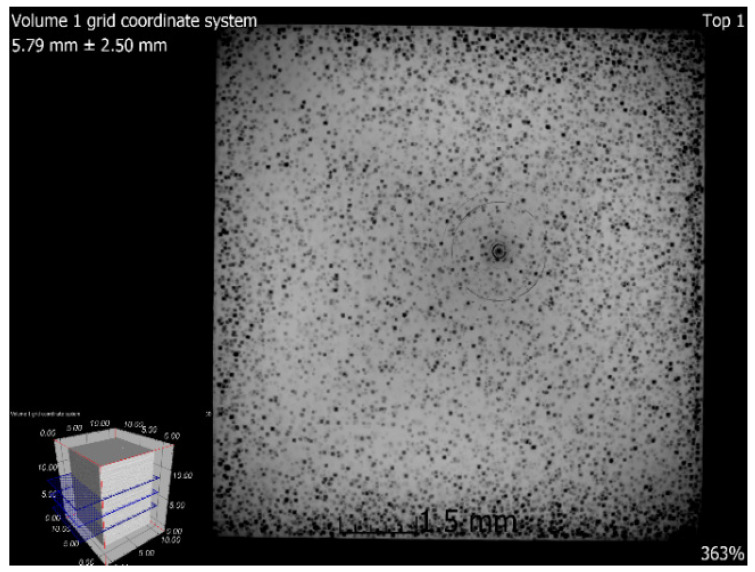
Tomographic CT sections of sample 5 after SLM (XY plane).

**Figure 17:**
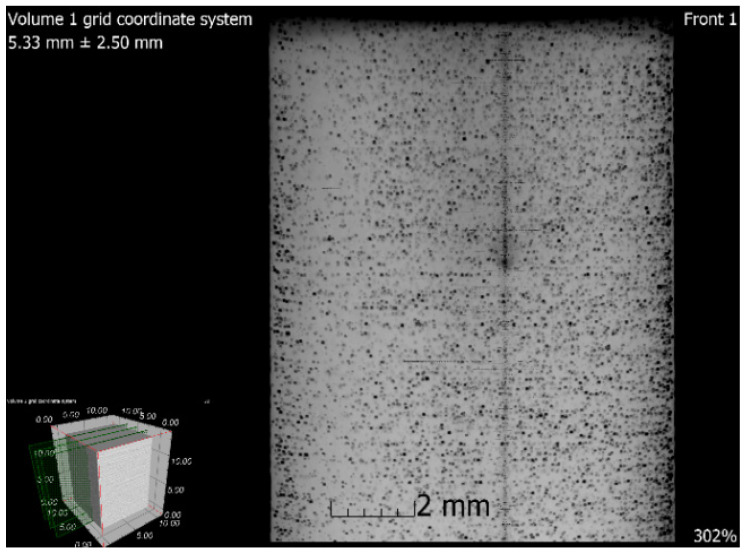
Tomographic CT sections of sample 5 after SLM (XZ plane).

**Figure 18:**
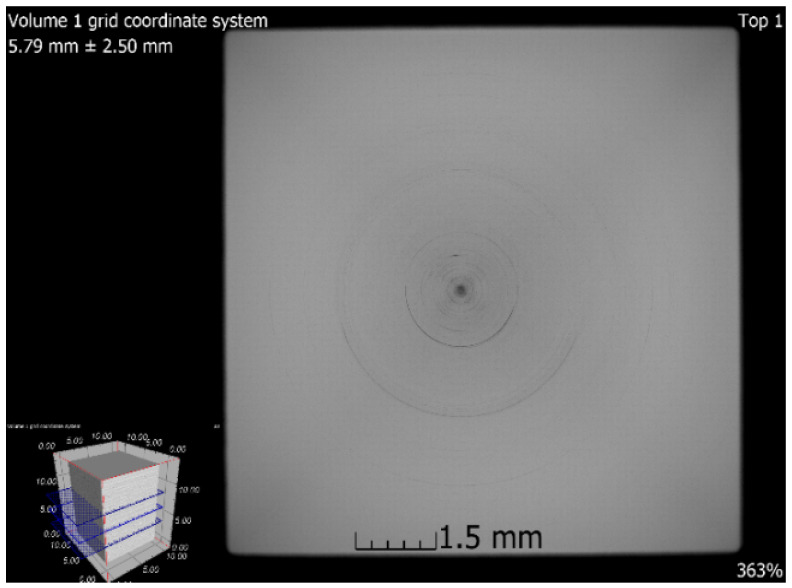
Tomographic CT sections of sample 5 after SLM + HIP_2 (XY plane).

**Figure 19:**
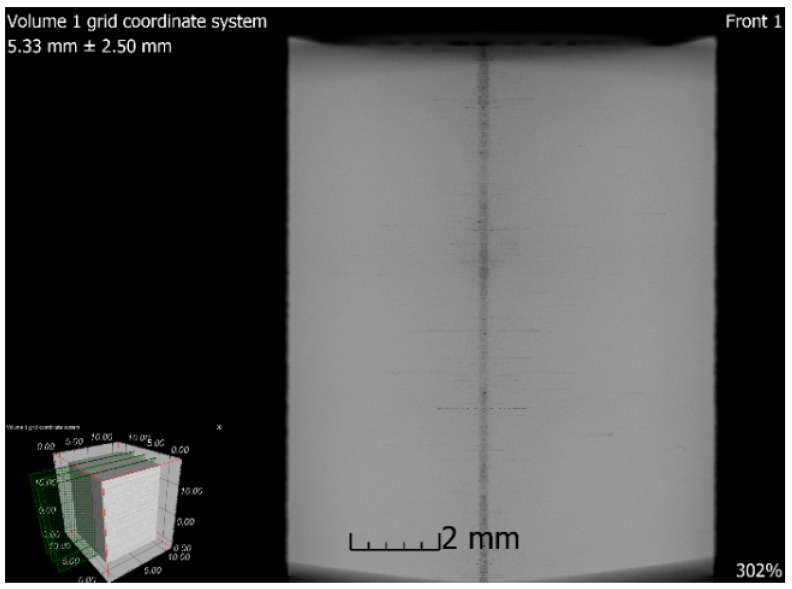
Tomographic CT sections of sample 5 after SLM + HIP_2 (XZ plane).

**Figure 20:**
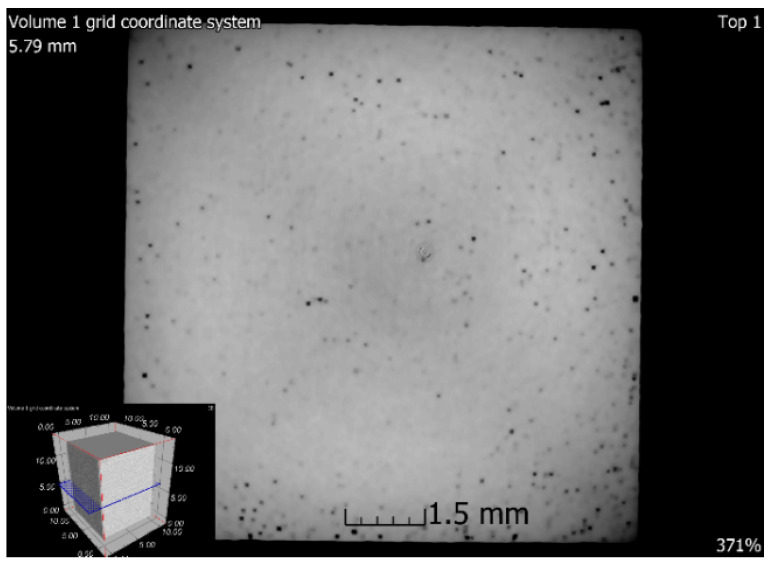
Tomographic CT sections of sample 5 with an adaptive Gaussian filter.

**Figure 21:**
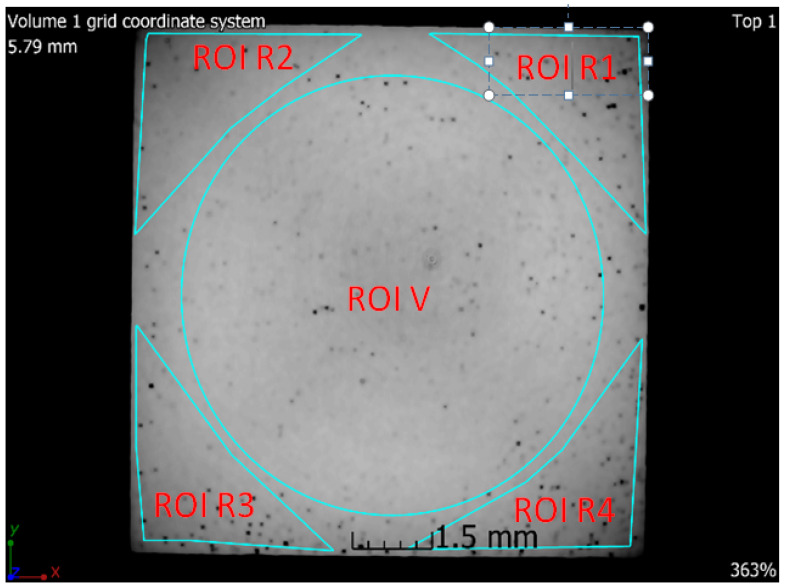
Tomographic CT sections of sample 5 with marked ROIs (regions of interest).

**Figure 22:**
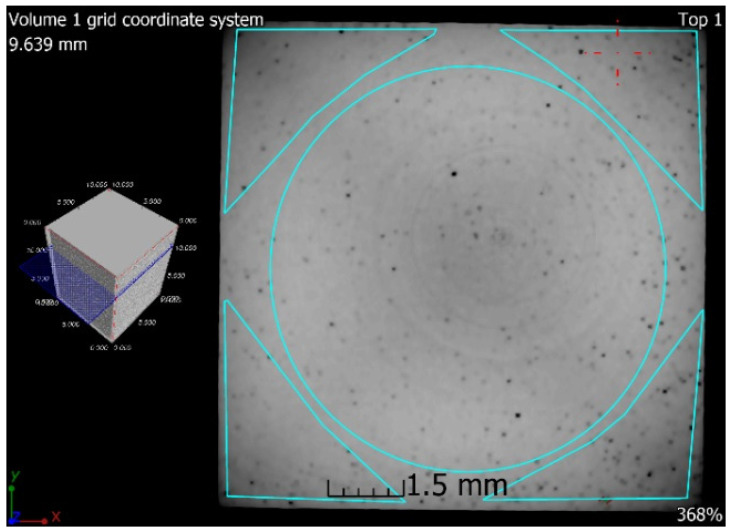
Segmentation and pore distribution in sample 5 after SLM.

**Figure 23:**
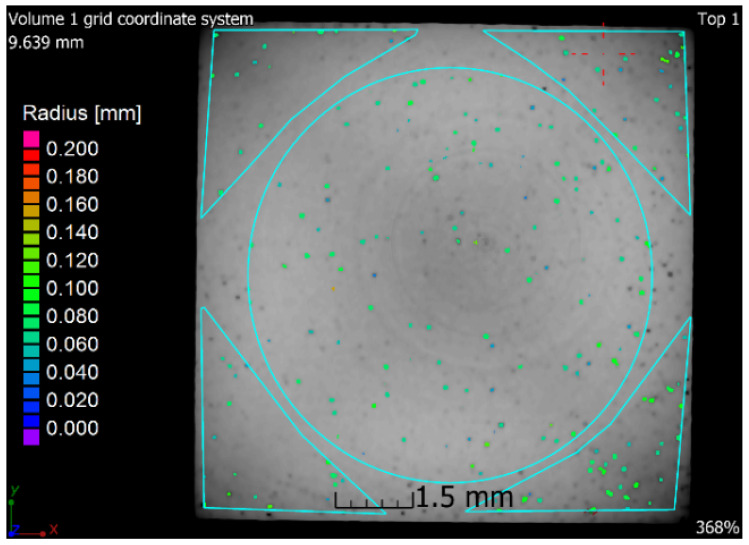
Example of detected pores in sample 5 after SLM.

**Figure 24:**
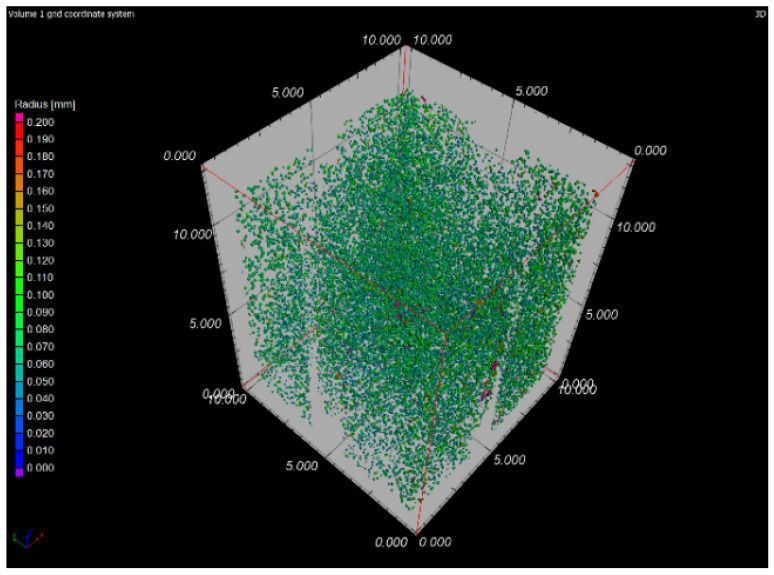
3D illustration of pore distribution in sample 5 after SLM.

**Figure 25:**
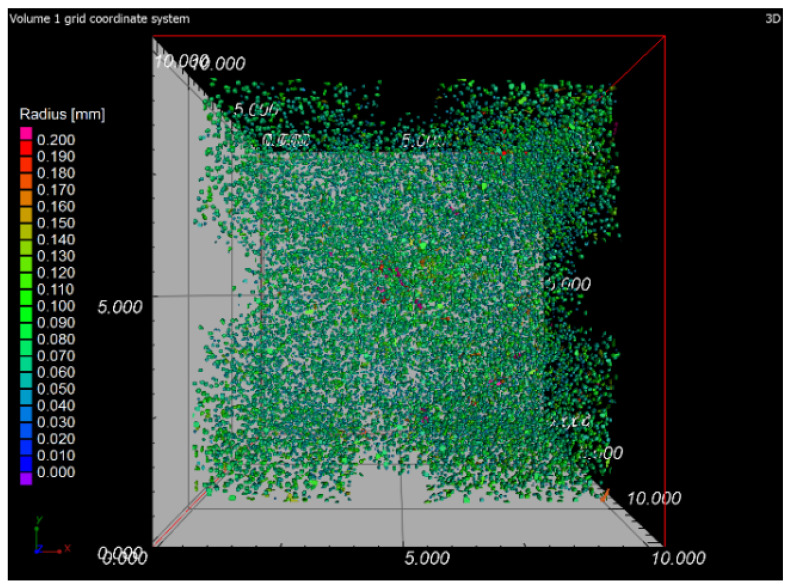
Pore distribution in the z direction (XY plane).

The samples 5–SLM and 5–SLM + HIP_2 were selected for the computed microtomography. The results of visualization of the pore space of both samples are evident from the projection of 101 CT sections of the sample into one tomographic CT sections (height of the displayed part of the sample: 5 mm; see Figure 16 and Figure 17). From these designed tomographic sections, the effect of the used HIP technology for the 3D-printed steel sample is obvious. No visible pores were found in the HIP sample; only the centers and ring artefacts caused by different sensitivities of individual pixels on the detector during their scanning are visible (see Figure 18 and Figure 19). The test specimen after SLM shows an uneven distribution of isolated pores with a higher density at the edges and corners of the test specimen. Before the actual porosity analysis, we used an adaptive Gaussian filter to reduce noise in tomographic sections. This filter allows blurring of the selected data set without destroying relevant structures like edges [26] (see Figure 20). Due to the uneven distribution of the gray level of individual voxels in the tomographic volume caused by both beam hardening effects and the orthogonal cross-section of the samples, we analyzed the pores in five regions of interest (ROIs), R1, R2, R3, R4 and V, for more accurate segmentation (see Figure 21). The “Porosity/Inclusion Analysis” module and its algorithm “2” were used for segmentation and analysis of the pore space [26]. Based on the measurement of pore size on unfiltered tomographic sections and due to the quality of tomographic data, a restriction concerning the size of their volume was used in the applied segmentation algorithm in its “Size range section” setting. The minimum defect (pore) volume was set to 70 Vxs, i.e., for pores with a volume of approx. 3.6 × 10^−8^ cm^3^ and a pore size of approx. 0.03 mm. Due to the nature of CT data, the value for min. volume should be no less than 8 voxels in edge length (i.e., an area of 2 × 2 × 2 voxels). It was necessary increased the value for lower CT data quality [26]. The maximum defect (pore) volume was set to 2000 Vxs, i.e., for pores with a volume of about 1 × 10^−6^ cm^3^ and a pore size of about 0.15 mm. An illustrative result of segmentation and pore distribution in individual ROIs is shown in Figure 22, Figure 23, Figure 24 and Figure 25. The values of porosity in an individual ROI’s range of about 0.9–2.5% are listed in Table 6, from which it is clear that the volume of pores in ROI R1 and R4 is about 2.4 times higher, and in ROI R2 and R3, it is about 1.4 times higher than in region V, which is located in the middle of the sample. The computed tomography values of porosity found for sample 5 SLM (0.88–2.2%) are in good agreement with the closed porosity values obtained by density measurements of sample 5 (1.21%) and with the detected values obtained by the image analysis (0.69%).

### 3.7. Tensile Testing

**Figure 26:**
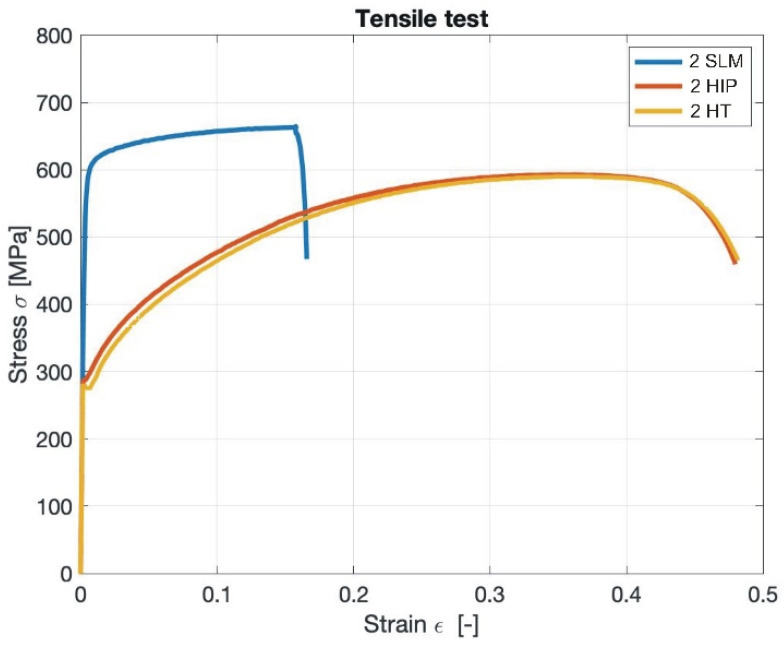
Stress–strain curves for sample 2 after SLM, SLM + HIP_2 and SLM + heat treated (HT).

**Figure 27:**
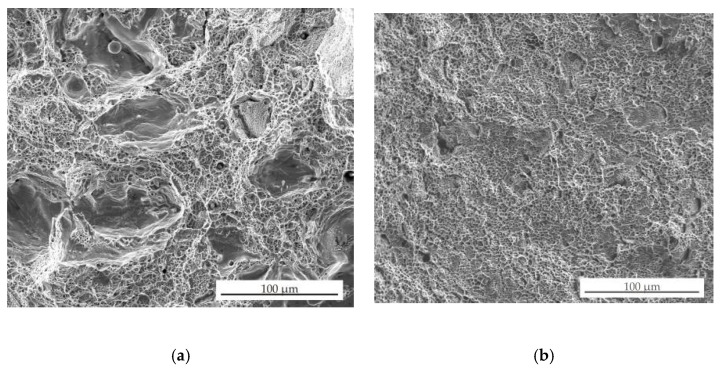
Fracture surfaces of sample (**a**) 13 after SLM and (**b**) 13 after SLM + HIP_2 (SEM/SE).

Figure 26 shows an example of strain–stress curves for sample 2 after SLM, HIP_2 and HT. Table 7 states the determined mechanical properties of samples after SLM, SLM + HIP 1125 °C/4 h/190 MPa and SLM + HT 1125 °C/4 h, respectively. The mechanical properties for samples 2 and 13 after SLM and SLM + HIP obtained from this work were compared with other similar works employing HIP to SLM stainless steel 316 L, as shown in Table 8. For SLM samples, relatively high values of yield strength were found. For SLM samples 1, 2, 5, and 6, in which the internal closed porosity was not higher than 1.3%, the yield strength reached values higher than 560 MPa. For samples 9 and 13, which contained higher levels of internal closed porosity, the yield strength reached lower values of around 500 MPa. The high yield strength values of SLM samples can be explained by their fine-grained structure [2]. However, the elongation of the samples after SLM was relatively low and reached values of 30–35%. In addition, some samples (5, 6, 9, and 13) failed outside the measured area, indicating the presence of internal porosity. The presence of internal porosity confirmed Figure 27a, which shows fracture surface of sample 13 after SLM. The powder residues inside the pores suggested that this is a LOF porosity, despite their round shape and use of a higher laser power. The comparison of the mechanical properties of samples prepared by SLM under the stated conditions revealed that both yield strength and tensile strength reached higher values than Lavery, Rottger or Chadha [2,4,12] and also higher ductility. However, the ductility did not reach as high values (76%) as Puichaud states for samples of 316 L steel prepared by SLM [17]. However, it must be taken into account that the samples prepared in the context of our study were built in the vertical direction, which, as some publications state [2,4,12], is not as suitable in terms of mechanical properties as the horizontal direction. Therefore, it can be assumed that with use of the horizontal direction of preparation, the values of mechanical properties would be even higher.

Samples after HIP, on the other hand, show lower yield strengths than after SLM (from 290 to 325 MPa), but also relatively high ductility of 47.8–48.5%, regardless on the used SLM conditions. Furthermore, in all samples after HIP, there was no failing outside of the measured area. The fracture surfaces of samples after HIP show the typical fracture with strong necking and without significant porosity (see Figure 27b). Lower yield strength for samples after HIP can be explained by larger grain size. Both the Young modulus and the tensile strength show similar values as the cast 316 L material as published by Rottger [4], 200 GPa and 600 MPa, respectively. Yield strength for HIP samples was a little lower than that published for cast material: 300 versus 365 MPa, and also ductility of 48% versus 70%, respectively. However, the determined values of yield strength and tensile strength for samples after HIP at 1125 °C/ 4 h/190 MPa are mostly higher than values published by Lavery [2] or Saiedi [27], who state for samples after SLM and HIP yield strength values of 227 and 220 MPa and tensile strength of 542 and 570 MPa, respectively, with similar or better ductility (41% for Lavery and 54% for Saiedi).

Samples after SLM and HT showed values of yield strength slightly lower than for SLM + HIP and also similar ductility values for samples with internal closed porosity of up to 1.3%, which indicates a significant effect of changes in the microstructure during heat treatment (the same temperature and dwell time as those during HIP were used). However, for samples 9 and 13, which had an internal porosity content equal or higher than 1.5%, there was a significant reduction in ductility during some tensile tests, and some failures also occurred outside of the measured area. From the performed tensile tests, it can be concluded that HIP had a very good effect on the stabilization of mechanical properties of samples after SLM, which were prepared by different parameters of 3D printing, and it reduced deviations between individual samples.

## 4. Conclusions

The effect of hot isostatic pressing on porosity and mechanical properties of 316 L stainless steel prepared by the selective laser melting method were studied. The following conclusions were reached:

1. The samples after SLM contained different contents of the closed internal porosity (measured by the pycnometric method) from 0.2 to 2%.
2. Scanning speed has the highest effect on the content of the porosity. With use of a scanning speed of 1200 mm/s, the high contents of open interconnected LOF porosity (up to 18%, measured by image analysis) were observed. The effect of a higher scanning speed is not so obvious when using higher laser powers of about 300–350 W.
3. The X-ray-computed microtomography and optical image analysis revealed that the samples after SLM contain higher proportions of porosity at the edges and corners than in the middle part.
4. Hot isostatic pressing at 1125 °C/4 h/137 MPa reduced the internal closed porosity to below 0.25%. For the samples with higher levels of open porosity (prepared with using of scanning speed 1200 mm/s), the hot isostatic pressing is not effective.
5. Hot isostatic pressing at 1125 °C/4 h/190 MPa reduced the internal closed porosity to below 0.11%.
6. Samples prepared by SLM show a relatively high yield strength of over 560 MPa and a high tensile strength of about 650 MPa, but only if the contents of porosity are not higher than 1.3%. If the content of porosity is higher than 1.3%, the samples show a lower yield strength and tensile strength of 500 and 600 MPa, respectively.
7. Samples after HIP show lower yield strengths than after SLM (from 290 to 325 MPa) and relatively high ductility of 47.8–48.5%, regardless of the used SLM conditions. HIP also reduces deviations between individual samples from SLM.

## Figures and Tables

**Table 1:** Conditions used for selective laser melting.

Sample Number	Set Power (W)	Scanning Speed (mm/s)	Exposure Time (µs)	Scan Strategy
1	200	400	138	Meander
2	200	650	80	Meander
3	200	800	63	Chessboard
4	200	1200	38	Chessboard
5	250	400	138	Meander
6	250	650	80	Meander
7	250	800	63	Chessboard
8	250	1200	38	Chessboard
9	300	400	138	Chessboard
10	300	650	80	Chessboard
11	300	800	63	Meander
12	300	1200	38	Meander
13	350	400	138	Chessboard
14	350	650	80	Chessboard
15	350	800	63	Meander
16	350	1200	38	Meander

**Table 2:** Results of density measurements and porosity levels in samples after SLM.

Sample	ρ_pycno_	V_pycno_	P_closed_	P_image_
	(g/cm^3^)	(cm^3^)	(%)	(%)
1	7.88 ± 0.01	3.12 ± 0.03	0.88 ± 0.21	0.49 ± 0.14
2	7.93 ± 0.01	3.16 ± 0.02	0.22 ± 0.09	0.13 ± 0.03
3	7.83 ± 0.01	3.17 ± 0.01	1.49 ± 0.08	1.12 ± 0.29
4	7.93 ± 0.02	2.55 ± 0.14	0.31 ± 0.11	18.70 ± 4.26
5	7.85 ± 0.02	3.09 ± 0.02	1.21 ± 0.21	0.69 ± 0.32
6	7.93 ± 0.01	3.16 ± 0.01	0.26 ± 0.09	0.22 ± 0.05
7	7.92 ± 0.01	3.17 ± 0.02	0.38 ± 0.14	0.37 ± 0.11
8	7.91 ± 0.01	2.74 ± 0.13	0.52 ± 0.13	15.31 ± 2.12
9	7.83 ± 0.02	3.05 ± 0.02	1.45 ± 0.19	1.46 ± 0.84
10	7.93 ± 0.01	3.12 ± 0.02	0.30 ± 0.10	0.31 ± 0.16
11	7.93 ± 0.02	3.16 ± 0.02	0.26 ± 0.09	0.29 ± 0.09
12	7.90 ± 0.02	2.95 ± 0.08	0.65 ± 0.18	8.78 ± 1.30
13	7.79 ± 0.02	3.04 ± 0.02	2.04 ± 0.39	1.54 ± 0.39
14	7.92 ± 0.01	3.08 ± 0.01	0.33 ± 0.09	0.30 ± 0.11
15	7.92 ± 0.02	3.15 ± 0.02	0.38 ± 0.08	0.29 ± 0.13
16	7.83 ± 0.02	3.06 ± 0.09	1.45 ± 0.31	5.12 ± 1.39

**Table 3:** Chemical composition of used powder.

Element	Declared Chemical Composition of the Manufacturer (wt.%)	Measured Chemical Composition (wt.%)
Fe	Bal.	Bal.
Cr	16–18	18.3 ± 0.5
Ni	10–14	11.2 ± 1.2
Mo	2–3	2.2 ± 0.1
Mn	≤2	1.9 ± 0.2
Si	≤1	1.1 ± 0.2
O	≤0.1	0.05 ± 0.01
N	≤0.1	0.03 ± 0.01
C	≤0.03	0.02 ± 0.01
S	≤0.03	0.004 ± 0.001

**Table 4:** Determined porosity levels in the samples after SLM and SLM + HIP_1 (porosity of HIP samples is determined on the basis of one measurement of a rectangular sample).

	After SLM		After HIP_1	
Sample	P_closed_	P_image_	P_closed_	P_image_
	(%)	(%)	(%)	(%)
1	0.88 ± 0.21	0.49 ± 0.14	0.13 ± 0.01	0.03 ± 0.01
2	0.22 ± 0.09	0.13 ± 0.03	0.15 ± 0.01	0.03 ± 0.02
3	1.49 ± 0.08	1.12 ± 0.29	0.25 ± 0.01	0.04 ± 0.02
4	0.31 ± 0.11	18.70 ± 4.26	0.57 ± 0.01	19.31 ± 5.12
5	1.21 ± 0.21	0.69 ± 0.32	0.16 ± 0.01	0.05 ± 0.01
6	0.26 ± 0.09	0.22 ± 0.05	0.18 ± 0.01	0.05 ± 0.02
7	0.38 ± 0.14	0.37 ± 0.11	0.11 ± 0.01	0.14 ± 0.03
8	0.52 ± 0.13	15.31 ± 2.12	0.33 ± 0.01	15.91 ± 3.39
9	1.45 ± 0.19	1.46 ± 0.84	0.16 ± 0.01	0.11 ± 0.08
10	0.30 ± 0.10	0.31 ± 0.16	0.16 ± 0.01	0.09 ± 0.04
11	0.26 ± 0.09	0.29 ± 0.09	0.19 ± 0.01	0.06 ± 0.03
12	0.65 ± 0.18	8.78 ± 1.30	0.43 ± 0.01	7.44 ± 1.91
13	2.04 ± 0.39	1.54 ± 0.39	0.16 ± 0.01	0.04 ± 0.01
14	0.33 ± 0.09	0.30 ± 0.11	0.14 ± 0.01	0.16 ± 0.08
15	0.38 ± 0.08	0.29 ± 0.13	0.15 ± 0.01	0.09 ± 0.06
16	1.45 ± 0.31	5.12 ± 1.39	0.98 ± 0.01	4.17 ± 1.96

**Table 5:** Determined porosity levels in the samples after SLM and SLM + HIP_1 and SLM + HIP_2 (porosity of samples after the HIP_2 process is determined on the basis of one measurement of rectangular samples and three measurements of cylindrical samples, a total of four).

Sample	ρ _pycno_	P_closed_
	(g/cm^3^)	(%)
13	7.79 ± 0.02	2.04 ± 0.39
HIP_1	7.94 ± 0.001	0.16 ± 0.01
HIP_2	7.94 ± 0.001	0.08 ± 0.02
9	7.83 ± 0.02	1.45 ± 0.19
HIP_1	7.94 ± 0.001	0.16 ± 0.01
HIP_2	7.95 ± 0.001	0.06 ± 0.03
5	7.85 ± 0.02	1.21 ± 0.21
HIP_1	7.94 ± 0.001	0.16 ± 0.01
HIP_2	7.95 ± 0.001	0.11± 0.03
6	7.93 ± 0.01	0.26 ± 0.09
HIP_1	7.94 ± 0.001	0.18 ± 0.01
HIP_2	7.94 ± 0.001	0.06 ± 0.01
1	7.88 ± 0.01	0.88 ± 0.21
HIP_1	7.94 ± 0.001	0.13± 0.01
HIP_2	7.94 ± 0.001	0.03 ± 0.02
2	7.93 ± 0.01	0.22 ± 0.09
HIP_1	7.93 ± 0.001	0.15± 0.01
HIP_2	7.95 ± 0.001	0.08 ± 0.03

**Table 6:** The values of porosity for sample 5 SLM in individual regions of interest.

ROI	Porosity P	Porosity Ratio (ROIR1–R4;V/ROIV)	Number of Defects (Pores)
	(%)	(--)	(1/cm^3^)
R1	2.06	2.3	141,278
R2	1.02	1.2	61,576
R3	1.39	1.6	90,710
R4	2.20	2.5	136,918
V	0.88	1.0	69,536

**Table 7:** Tensile properties for 316L steels after SLM, SLM + HIP_2 and SLM + HT (X indicates that some tensile specimens have failed outside the measured area).

Sample	Young Modulus (GPa)	Yield Strength (MPa)	Tensile Strength (MPa)	Elongation at Failure (%)
1	204 ± 1	573 ± 2	639 ± 1	30.4 ± 4
2	206 ± 1	575 ± 4	662 ± 2	32.05 ± 0.35
5	207 ± 1	572 ± 3	660 ± 3	34.2 ± 0.2 X
6	209 ± 3	564 X	653 X	34.1 X
9	245 ± 37	504 ± 12	602 ± 26	34.5 X
13	187 ± 20	511 ± 8	612 ± 16	32.7 X
1 HIP	211 ± 32	321 ± 12	578 ± 2	48.5 ± 0.1
2 HIP	195 ± 5	292 ± 4	595 ± 3	48.1 ± 0.3
5 HIP	208 ± 5	292 ± 3	595 ± 1	47.8 ± 0.2
6 HIP	192 ± 9	291 ± 2	549 ± 43	47.8 ± 0.4
9 HIP	210 ± 12	325 ± 9	585 ± 2	47.8 ± 1.3
13 HIP	198 ± 2	320 ± 9	585 ± 2	48.5 ± 0.1
1 HT	212 ± 15	277 ± 3	569 ± 2	48.1 ± 0.4
2 HT	191 ± 1	276 ± 1	589 ± 2	48.1 ± 0.4
5 HT	207 ± 23	276 ± 2	587 ± 1	50.1 ± 0.6
6 HT	177 ± 15	276 ± 2	587 ± 3	48.7 ± 0.3
9 HT	154 ± 52	288 ± 4	578 ± 3	42.7 ± 5.3 X
13 HT	183 ± 9	285 ± 1	571 ± 9	42.5 X

**Table 8:** Results of tensile experiments and values of mechanical properties of SLM and HIP samples available in the literature.

Sample	Young Modulus	Yield Strength	Tensile Strength	Elongation to Fracture	HIP Conditions	Porosity	SLM—Scanning Speed/Power
-	(Gpa)	(Mpa)	(Mpa)	(%)	(temperature/ time/pressure)	(%)	(mm/s)/(W)
Rottger 75/30 [4]	165	438	528	10	-	2.8	400/100
Rottger 250/100 [4]	145	406	510	18	-	2	400/100
Lavery SLM [2]	196	385	524	22	-	2	600/180
Puichaud SLM [17]	-	520	580	76	-	0.1	675/150
Chadha SLM [12]	-	445	585	21	-	0.4	1000/350
This work sample 2 SLM	206	575	662	32	-	0.2	650/200
This work sample 13 SLM	187	511	612	33	-	2	400/350
Cast sample [4]	200	365	596	69	-	0	-
Saiedi [27]	-	220	570	54	-	0.3	800/190
Rottger 250/100 HIP [4]	171	201	428	38	1150/3/150	4.7	400/100
Lavery SLM HIP [2]	202	227	542	41	1125/4/137	1.1	600/180
Puichaud SLM HIP [17]	-	260	570	80	1100/3/180	0	675/150
Chadha SLM HIP [12]	-	263	611	48	1163/3/100	0.2	1000/350
This work sample 2 SLM + HIP	195	292	595	48	1125/4/190	0.08	650/200
This work sample 13 SLM + HIP	198	320	585	49	1125/4/190	0.08	400/350
